# CpG oligonucleotides suppress HepG2 cells-induced Jurkat cell apoptosis *via *the Fas-FasL-mediated pathway

**DOI:** 10.1186/1756-9966-30-48

**Published:** 2011-05-03

**Authors:** Jianfeng Zheng, Rongquan Fu, Jing Li, Xiaozhong Wang

**Affiliations:** 1 Department of Clinical Laboratory, the Second Affiliated hospital of Nanchang University, Nanchang 330006, China; 2 Department of Infectious Diseases, the Third Affiliated Hospital of Wenzhou Medical College, Rui'an 325200, China; 3 Department of Clinical Laboratory, the First Affiliated hospital of Nanchang University, Nanchang 330006, China

**Keywords:** CpG-ODN, hepatocellular carcinoma, apoptosis

## Abstract

**Objective:**

To explore the potential role of CpG motif-containing oligonucleotides (CpG-ODN) in modulating the expression of FasL in HepG2 and Fas in Jurkat cells *in vitro*, and to examine the effect of CpG-ODN treatment on the HepG2 cells-mediated Jurkat cell apoptosis *in vitro*.

**Methods:**

The expressions of FasL in HepG2 and Fas in Jurkat cells were examined by real time PCR and flow cytometry (FCM). HepG2 and Jurkat cells were co-cultured, and the frequency of apoptotic Jurkat cells and levels of activated caspase-3 were determined by FCM.

**Results:**

Treatment with CpG-ODN down-regulated the expression of FasL in HepG2 cells in a dose- and time-dependent manner. In addition, treatment with CpG-ODN down-regulated the Fas mRNA transcription and protein expression in Jurkat cells. Treatment of HepG2 cells or Jurkat cells with FasL-neutralizing antibody NOK-2 remarkably inhibited the HepG2-medaited Jurkat cell apoptosis. Pre-treatment of HepG2 or Jurkat cells with CpG-ODN significantly reduced the frequency of HepG2-mediated apoptotic Jurkat cells and inhibited the activation of caspase-3 in Jurkat cells *in vitro*.

**Conclusions:**

Our data indicated that treatment with CpG-ODN inhibited the HepG2 cells-mediated Jurkat cell apoptosis by modulating the Fas/FasL pathway. Apparently, CpG-ODN treatment may be a potential therapeutic reagent for HCC.

## Introduction

Tumors escape immune surveillance through multiple mechanisms. For example, tumors can produce inhibitory factors, such as transforming growth factor-β (TGF-β) and vascular endothelial growth factor (VEGF), leading to the reduced dendritic cell activation and impaired tumor-specific T cell immunity [1]. Tumor cells can up-regulate some of the functional surface molecules, including FasL, which can actively induce the apoptosis of the Fas-expressing activated T lymphocytes, while others can down-regulate the expression of other molecules, such as MHC class I and Fas [2,3]. Although the mechanisms by which tumor cells evade immune surveillance are not well understood, the selective induction of tumor cell apoptosis has been thought to be a valuable strategy for tumor therapy. CpG-ODN can function as a Th-1 adjuvant [4] and is able to activate dendritic cells [5]. Accordingly, CpG-ODN has been used as an adjuvant for the induction of anti-tumor immune responses [6-8].

Hepatocellular carcinoma (HCC) is one of the most common malignant tumors worldwide, particularly in China. Accumulating evidences have suggested that several mechanisms contribute to the carcinogenesis of HCC [9,10]. The relative resistance to apoptosis triggering and the strong proliferation in HCC cells have been thought as predominant factors contributing to the development of HCC [11]. Recently, high levels of FasL have been found in HCC tumor cells [12]. Given that Fas is highly expressed by activated T cells, HCC may trigger the apoptosis of activated T cells through the Fas/FasL pathway, escaping from immune surveillance. However, little is known whether CpG-ODN could modulate the expression of FasL in HCC cells and Fas in human T cells as well as the HCC-triggered human T cell apoptosis.

This study aimed at exploring the potential effect of CpG-OND treatment on the HepG2-induced Jurkat cell apoptosis. We found that treatment with CpG-ODN down-regulated the expression of FasL in HepG2 cells and Fas in Jurkat cells, and inhibited the HepG2-mediated Jurkat cell apoptosis in vitro. We discussed the implication of our findings.

## Materials & methods

### Reagents

The CpG-ODN-M362 [13] used in the experiment was synthesized by Invitrogen (Invitrogen Inc, Shanghai, China). Oligonucleotides were dissolved in TE-buffer (pH 8.0) containing 10 mM Tris-HCl and 1 mM EDTA at a concentration of 100 μM, which were then aliquoted and stored at -20°C until use. RPMI-1640 medium was obtained from Invitrogen Inc. (Carlsbad, CA, USA). Fetal bovine serum (FBS) was purchased from GIBCO BRL (Grand Island, NY, USA). Monoclonal antibody against human FasL, NOK-2, was purchased from BD Pharmingen (San Diego, CA, USA).

### Cell culture

Human hepatocellular carcinoma cell line, HepG2 and lymphoma cell line, Jurkat were maintained in our laboratory and cultured in RPMI-1640 medium supplemented with 10% FBS, 100 U/mL penicillin, and 100 μg/mL streptomycin in 25 cm^2 ^polystyrene flasks at 37°C in a humidified atmosphere of 5% CO_2 _incubator. Routine passage was carried out every 2 or 3 days.

### Flow cytometry analysis

HepG2 cells at 5 × 10^5 ^cells/well were treated in duplicate with 10^-4 ^to 5 μM CpG-ODN in 10% FBS RPMI1640 in 12-well plates for 48 h to determine the optimal dosage of CpG-ODN for modulating the FasL expression. In addition, HepG2 cells at 5 × 10^5 ^cells/well were treated in duplicate with 1 μM CpG-ODN for 0-48 h. The cells were harvested and stained with phycoerythrin (PE) anti-human FasL antibody and isotype control (eBioscience, San Diego, CA, USA). The frequency of Fas-expressing HepG2 cells were determined by flow cytometry analysis. Approximately, 10,000 cells from each sample were analyzed by flow cytometry on a FACS Calibur instrument (Becton Dickinson, San Jose, CA, USA).

Jurkat cells at 5 × 10^5 ^cells/well were treated in duplicate with 1 μM CpG-ODN for 24 h and cultured in medium alone as controls. The cells were harvested and stained with PE-anti-human Fas antibody or isotype control (eBioscience). The frequency of Fas-expressing cells was determined by flow cytometry analysis. Data were analyzed using CellQuest software.

### HepG2 and Jurkat cells coculture

HepG2 cells at 2 × 10^6 ^cells/well were cultured in 10% FBS RPMI1640 alone or treated with 1 μM CpG-ODN or 10 μg/ml anti-FasL antibody NOK-2 in RPMI1640 for 24 h to prepare the inducers. Jurkat cells at 2 × 10^6 ^cells/well were cultured 10% FBS RPMI1640 alone or treated with 1 μM CpG-ODN or 10 μg/ml anti-FasL antibody NOK-2 in RPMI1640 for 24 h to prepare the target cells. These cells were cultured as the untreated HepG2 (2 × 10^6^) and Jurkat cells (4 × 10^5^) for 24 h (controls); the NOK-2-treated HepG2 and untreated Jurkat cells; the untreated HepG2 and the NOK-2-treated Jurkat cells; the CpG-ODN-treated HepG2 and untreated Jurkat cells; and the untreated HepG2 and the CpG-ODN-treated Jurkat cells, respectively. Subsequently, the suspended Jurkat cells were collected and stained with FITC-Annexin V and PI. The apoptotic Jurkat cells were determined by flow cytometry analysis. Data were analyzed using CellQuest software.

In addition, the unmanipulated Jurkat cells or the CpG-ODN-treated Jurkat cells were harvested after co-culture with unmanipulated HepG2 or the CpG-ODN-treated HepG2 cells. The cells were stained with PE-anti-activated caspase-3 using the PE-conjugated active caspase-3 apoptosis kit (BD Pharmingen), and the activation of capsase-3 was determined by flow cytometry analysis.

### qRT-PCR

Total RNA was extracted from the unmanipulated and CpG-ODN-treated Jurkat cells using Trizol reagent, according to the manufacturer's instructions (Invitrogen, Carlsbad, CA, USA), and reversely transcribed into cDNA using oligo (dT) 12-18 and ReverTraAce-α™ (Toyobo. Co., Japan), resepctively. The relative levels of Fas mRNA transcripts to control GAPDH were determined by quantitative real-time PCR using the SYBR Green One-Step kit and the specific primers on a LightCycler™ (Roche Diagnostics, Mannheim, Germany). The sequences of the primers were synthesized by Invitrogen (Invitrogen Inc, Shanghai, China) and are presented in Table 1. The PCR reactions containing 0.4 μM FasL primers, 2.5 μM MgCl_2_, 1 × SYBR Green master mix, and 1 μL cDNA were performed in duplicate at 95°C for 5 min for denaturation and subjected to 40 cycles of 95°C for 15 s, 57°C for 5 s, 72°C for 10 s and then 78°C for 5 s. Data were analyzed using LightCycler analysis software. The individual PCR efficiencies were determined using LinRegPCR [14], and the mRNA expressions (rER values) for Fas and FasL were calculated by the Gene Expression's C (T) Difference (GED) method [15].

**Table 1:** the sequences of primers.

Target gene	Primers	Annealing temperature (°C)
Fas	Forward:5'-AGCTTGGTCTAGAGTGAAAA-3' Reverse: 5'-GAGGCAGAATCATGAGATAT-3'	51
FasL	Forward: 5'-CACTTTGGGATTCTTTCCAT-3' Reverse: 5'-GTGAGTTGAGGAGCTACAGA-3'	57
GAPDH	Forward: 5'-GAAGGTGAAGGTCGGATGC-3' Reverse: 5'-GAAGATGGTGATGGGATTTC-3'	61

### Statistical analysis

Data were expressed as means ± S.E.M. Statistical significance was assessed using either Student's *t*-test or one-way ANOVA followed by *post hoc *Dunnett, SNK test. A value of *p *< 0.05 was considered significantly different.

## Results

### CpG-ODN downregulated the expression of FasL in HepG2 cells in a dose- and time-dependent manner

**Figure 1:**
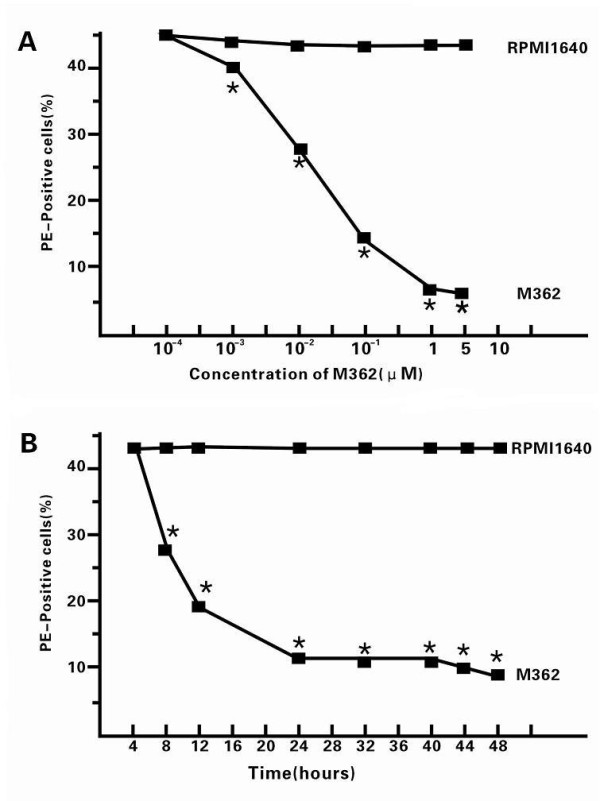
**Treatment with CpG-ODN inhibited the expression of FasL in HepG2 cells in a dose- and time-dependent manner**. (A) Dose effect. HepG2 cells were treated with different concentrations of CpG-ODN for 48 h. (B) Time effect. HepG2 cells were treated with 1 μM CpG-ODN for the indicated time periods. The cells were harvested, and the frequency of FasL-positive cells was determined by FACS analysis. Data are expressed as mean% ± SEM of each group of the cells from four independent experiments. *p < 0.05 vs. controls.

To determine the effect of CpG-ODN treatment on the expression of FasL, HepG2 cells were treated with various doses of CpG-ODN (10^-4^-5 μM) for 12 hours, and the frequency of FasL-positive cells was determined by flow cytometry analysis (Figure 1A). Treatment with the CpG-ODN at 10^-3 ^μM significantly reduced the frequency of FasL-expressing HepG2 cells, and treatment with increased doses of the CpG-ODN further decreased the frequency of FasL positive HepG2 cells *in vitro*. Furthermore, we found that the effects of treatment with 1 μM CpG-ODN on the expression of FasL in HepG2 cells were time-dependent. Evidentially, treatment with 1 μM CpG-ODN for 8 h reduced the frequency of FasL-expressing HepG2 cells to 28% and treatment for 24 h decreased the frequency of FasL-expressing HepG2 cells to near 10%. Apparently, treatment with CpG-ODN inhibited the expression of FasL in HepG2 cells in a dose- and time-dependent manner.

### Effect of CpG-ODN on the Fas expression in Jurkat cells

**Figure 2:**
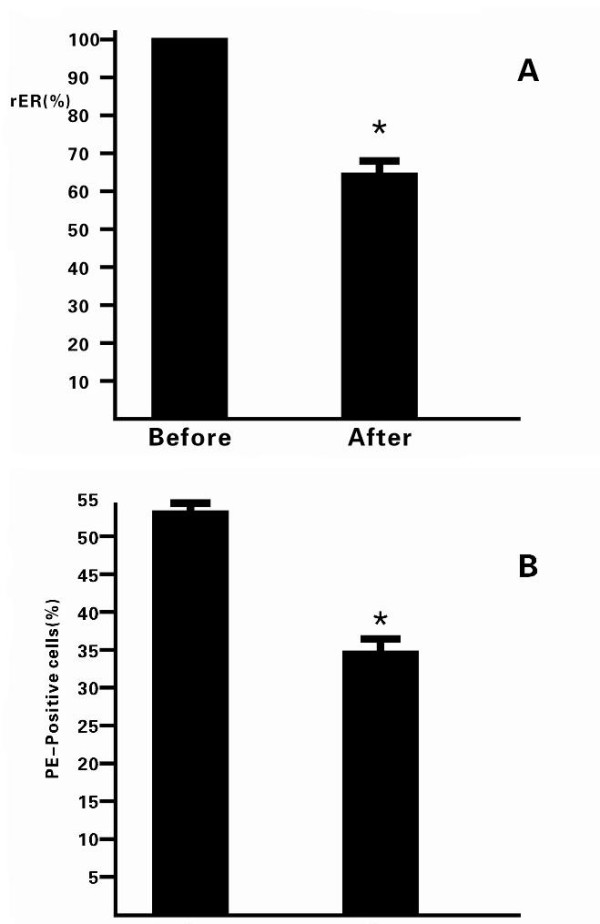
**Treatment with CpG-ODN inhibited the expression of Fas in Jurkat cells**. Jurkat cells were treated with 1 μM CpG-ODN for 24 h, and the cells were collected. The intracellular expression of Fas was examined by qRT-PCR (A) and FCM (B). Data are expressed as mean% ± SEM of each group of the cells from four separate experiments. *p < 0.05 vs. the controls.

Next, we tested whether treatment with CpG-ODN could modulate the expression of Fas in Jurkat cells. Jurkat cells were treated with 1 μM CpG-ODN for 24 h. The cells were harvested and the relative levels of Fas mRNA transcripts to control GAPDH were determined by quantitative RT-PCR (Figure 2A). Clearly, the relative levels of Fas mRNA transcripts in the CpG-ODN-treated Jurkat cells were reduced to 65%, as compared with that of unmanipulated controls. Furthermore, the expression of Fas in Jurkat cells was also examined by flow cytometry analysis. The frequency of Fas-expressing Jurkat cells was significantly reduced from 54% ± 2% to 35% ± 1% (Figure 2B). Therefore, CpG-ODN treatment down-regulated the Fas mRNA transcription and protein expression in Jurkat cells *in vitro*.

### Effect of CpG-ODN on the HepG2-mediated Jurkat cell apoptosis

**Figure 3:**
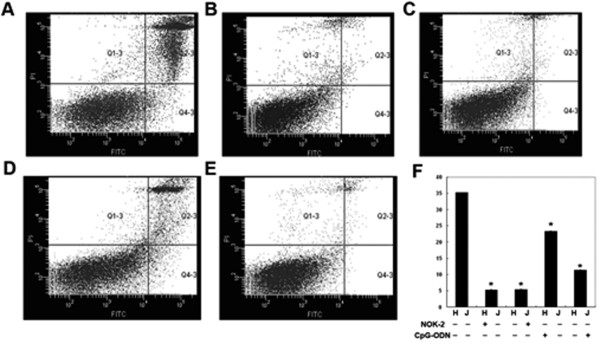
**Apoptosis of Jurkat cells induced by HepG2 cells**. HepG2 and Jurkat cells were cultured in medium alone or treated with 1 μM CpG-ODN or 10 μg/ml xx μg/ml anti-FasL NOK-2 antibody for 24 h. The cells were harvested and co-cultured as the unmanipulated HepG2 and Jurkat cells (A, positive controls), the NOK-2-treated HepG2 and unmanipulated Jurkat cells (B), the unmanipulated HepG2 and NOK_2-treated Jurkat cells (C), the CpG-ODN-treated HepG2 and unmanipulated Jurkat cells (D) or the unmanipulated HepG2 and CpG-ODN-treated Jurkat cells (E), respectively for 24 h. The unadhered Jurkat cells were harvested and stained with FITC-Annexin V and PI, followed by flow cytometry analysis. (F) Quantitative analysis. The frequency of apoptotic Jurkat cells was analyzed by using CellQuest software. Data are expressed as representative FCM or mean% ± S.E.M of each group of the cells from four independent experiments. *p < 0.05 vs. the positive controls.

Engagement of Fas on the cell membrane by FasL can trigger cell apoptosis. Given that CpG-ODN treatment down-regulated the expression of FasL in HepG2 cells and Fas in Jurkat cells, it is possible that CpG-ODN may modulate the HepG2 cell-mediated Jurkat cell apoptosis. Accordingly, we first treated HepG2 and Jurkat cells with 1 μM CpG-PDN or anti-FasL NOK-2 antibody for 24 h for the preparation of effector and target cells, respectively. Next, we co-cultured the unmanipulated HepG2 and Jurkat cells (positive controls), the NOK-2-treated HepG2 and untreated Jurkat cells, the untreated HepG2 and the NOK-2-treated Jurkat cells, the CpG-ODN-treated HepG2 and untreated Jurkat cells, and the untreated HepG2 and the CpG-ODN-treated Jurkat cells for 24, respectively. Subsequently, the suspended Jurkat cells were collected and the frequency of apoptotic Jurkat cells was determined by flow cytometry analysis (Figure 3). First, co-culture of HepG2 cells with Jurkat cells triggered Jurkat cell apoptosis (Figure 3A and 3F). Pre-treatment of either HepG2 or Jurkat cells with anti-FasL antibody significantly reduced the frequency of apoptotic Jurkat cells (Figure 3B and 3C), indicating that the FasL/Fas pathway might be involved in the apoptosis of Jurkat cells in this experimental system.

More interestingly, co-culture of the CpG-ODN-treated HepG2 cells with unmanipulated Jurkat cells or unmanipulated HepG2 with the CpG-ODN-treated Jurkat cells significantly reduced the frequency of apoptotic Jurkat cells, particularly following treatment of Jurkat cells with CpG-ODN. These data indicated that down-regulation of FasL and Fas expression by CpG-ODN in either HepG2 or Jurkat cells inhibited the HepG2 cell-mediated Jurkat cell apoptosis *in vitro*.

### Caspase-3 activity analysis

**Figure 4:**
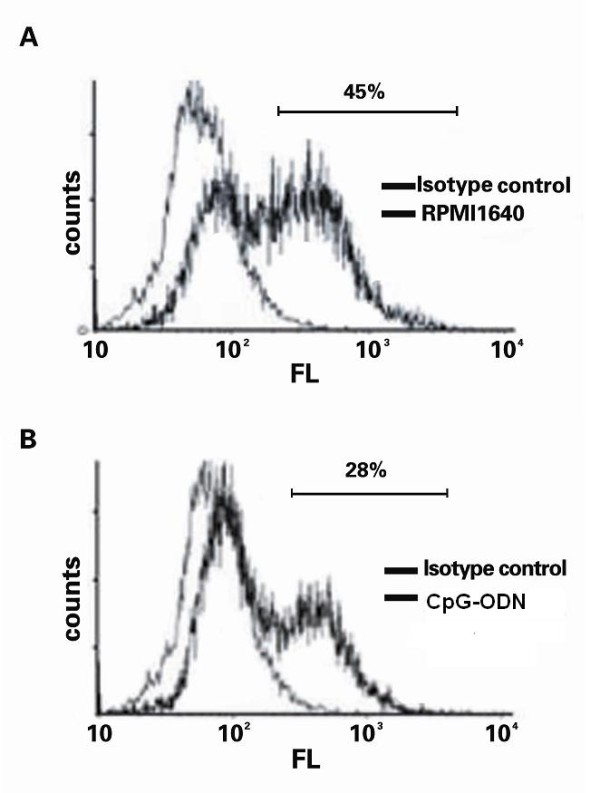
**CpG-ODN treatment suppressed the caspase-3 activation in Jurkat cells**. HepG2 and Jurkat cells were cultured in medium alone or treated with 1 μM CpG-ODN, respectively for 24 h. The unmanipulated HepG2 and Jurkat cells or the CpG-ODN-treated HepG2 and Jurkat cells were co-cultured for 24, respectively. The Jurkat cells were harvested and the contents of activated caspase-3 were determined by flow cytometry analysis. (A) The unmanipulated Jurkat cells; (B) The CpG-ODN-treated Jurkat cells. Data shown are representative histograms from each group of cells from four separate experiments. The percentage of positive cells was indicated.

The activation of caspase-3 is crucial for the intrinsic and extrinsic apoptotic pathways. Accordingly, we selectively examined the activity of caspase-3, a downstream factor of the Fas-FasL pathway. As shown in Figure 4, the levels of activated caspase-3 were significantly reduced in the CpG-ODN-treated Jurkat cells (28.20 ± 0.18%), as compared to unmanipulated Jurkat cells (45.15 ± 0.13%). These data suggested that the CpG-ODN reduced HepG2-induced Jurkat cell death through the caspase-3-dependent apoptotic pathway.

## Discussion

The up-regulated expression of FasL has been found in various types of tumors, including melanoma, lymphoma, gastric carcinoma, and breast carcinoma [16]. It has been reported that high levels of FasL expression are associated with the presence of tumor-infiltrating lymphocytes (TIL), leading to high susceptibility of activated T cells in tumor tissues to apoptosis triggers due to high levels of Fas expression by activated T cells [17]. Indeed, engagement of Fas by the FasL can promote the formation of death-inducing signaling complex, resulting in activated T cell apoptosis. This may partially contribute to tumor cells escaping from immune surveillance and leading to tumor progression.

Due to the important role of Fas in the tumor progression and metastasis, the Fas-mediated apoptosis might be a target for cancer therapy. Notably, the apoptotic cascade is a sequential process of many events that can be regulated at different stages. Several agents have been found to directly or indirectly inhibit cellular apoptosis. The arsenic trioxide and tumor necrosis factor-related apoptosis-inducing ligand receptor (TRAIL) can modulate the intrinsic and extrinsic pathways, respectively [18]. The caspase activators can regulate the common pathway, and ONY-015 can regulate modulators of the apoptosis pathways [19]. CpG-ODN can activate the nuclear factor kappa-light-chain-enhancer of activated B cells (NF-κB) and activated protein 1 through the Toll-like receptor (TLR) sigaling pathway [20], and has been thought to act as a potent adjuvant for inducing Th1 response. The NF-κB can regulate the expression of the FasL gene, exhibiting both anti-apoptotic and pro-apoptotic functions [19]. In this study, we examined the effects of CpG-ODN treatment on the HepG2 cell-induced Jurkat cell apoptosis. We found that CpG-ODN inhibited the expression of FasL in HepG2 in a dose- and time-dependent manner (Figure 1). Treatment with CpG-ODN at 1 μM for 24 h greatly inhibited the expression of FasL in HepG2 cells *in vitro*. Furthermore, we found that treatment with CpG-ODN effectively down-regulated the expression of Fas in human Jurkat cells (Figure 2). Jurkat cells are derived from human T lymphocyte leukemia cells, mimic the activated T lymphocyte cells, and have been widely used as experimental models to study the functions of T cells [21]. In addition, co-culturing the unmanipulated HepG2 cells with Jurkat cells triggered a high frequency of Jurkat cells undergoing apoptosis, which was effectively abrogated by pre-treatment of either HepG2 or Jurkat cells with anti-FasL antibody. These data indicated that HepG2 cells induced Jurkat cell apoptosis *via *the Fas/FasL pathway. More importantly, pre-treatment of Jurkat cells or HepG2 cells with CpG-ODN efficiently inhibited the HepG2-mediated Jurkat cell apoptosis (Figure 3) and the caspase activation in Jurkat cells (Figure 4). CpG-ODN can suppress apoptosis of macrophages via TLR9 through PKB/Akt/FOXO pathway [22], since macrophages and T cells play an important role in anti-tumor immune, our study showed CpG-ODN suppresses apoptosis through FasL/Fas pathway, maybe PKB/Akt/FOXO is another way in anti-apoptosis anti-cancer therapeutic strategies of CpG-ODN.

Currently, treatment of HCC relies on surgery, conventional chemotherapy, and radiation therapy at clinic. Other therapeutic strategies, such as an antibody targeting the specific molecules, are currently in trials. DNA-based drugs, such as CpG-ODN and antisense ODN, are regarded as a new alternative therapy for the brain tumors [23]. The regulation of the complex signaling pathways in tumors has been a new strategy for the rational design of anticancer strategies. Escaping from immune surveillance and being resistant to apoptosis triggers play an important role in the progression and metastasis of tumors. Our results indicated that CpG-ODN down-regulated the FasL expression in HepG2 cells and Fas in Jurkat cells, and suppressed the HepG2 cells-mediated caspase-dependent apoptosis of Jurkat cells. Conceivably, CpG-ODN treatment may be a promising strategy for the intervention of HCC.

## Competing interests

The authors declare that they have no competing interests.

## Authors' contributions

JZ carried out the molecular genetic studies; RF participated in the design of the study and performed the statistical analysis; JL participated in carried out the immunoassays; XW conceived of the study, and participated in its design and coordination and drafted the manuscript. All authors read and approved the final manuscript.
